# Community assembly and aroma contribution of *Hanseniaspora* yeasts in spontaneous fermentation of Cabernet Sauvignon grapes from four regions in Northwest China

**DOI:** 10.1016/j.fochx.2026.103899

**Published:** 2026-04-23

**Authors:** Boyuan Fan, Yuwei Chang, Mengni Zheng, Jinding Han, Yongsheng Tao, Hongyu Zhao, Kai Hu

**Affiliations:** a College of Enology, Northwest A&F University, Yangling, Shaanxi 712100, China; b Shaanxi Engineering Research Center for Viti-viniculture, Yangling, Shaanxi 712100, China

**Keywords:** *Hanseniaspora* yeast, Wine aroma, Spontaneous fermentation, Fungal diversity, *Terroir*, High-throughput sequencing, Yeast isolation

## Abstract

This study investigated the community assembly and aroma contribution of *Hanseniaspora* yeasts during spontaneous fermentation of Cabernet Sauvignon grapes from four regions (Zhangye, Wuhai, Yinchuan, and Xianyang) in Northwest China. The resulting wines were characterized by distinct profiles of volatile compositions and aroma characteristics. High-throughput sequencing identified *Hanseniaspora* as the most abundant non-*Saccharomyces* community during fermentation, while culture-dependent isolation of 138 *Hanseniaspora* strains showed differential community assembly of *H. uvarum*, *H. opuntiae*, *H. osmophila*, and *H. vineae* across regions. *H. osmophila* and *H. vineae* were notably enriched in high-sugar musts from Zhangye, Wuhai, and Yinchuan with high diurnal temperature range. Such enrichment may induce longer survival of *Hanseniaspora* community at later fermentation stage, giving wines higher levels of acetate ester and floral aroma. Partial least squares regression of *Hanseniaspora* species and volatile compounds further suggested the potential impact of *H. osmophila* on region-driven variation in acetate ester production.

## Highlights

• *Hanseniaspora* was the most abundant non-*Saccharomyces* yeast in all four regions.
• Culture-dependent methods revealed region-specific assembly of *Hanseniaspora* species.
• High-sugar musts from regions with high ADTR enriched *H. osmophila* and *H. vineae*.
• PLSR confirmed the strong correlation of *H. osmophila* with acetate esters.

## Graphical abstract

**Figure:**
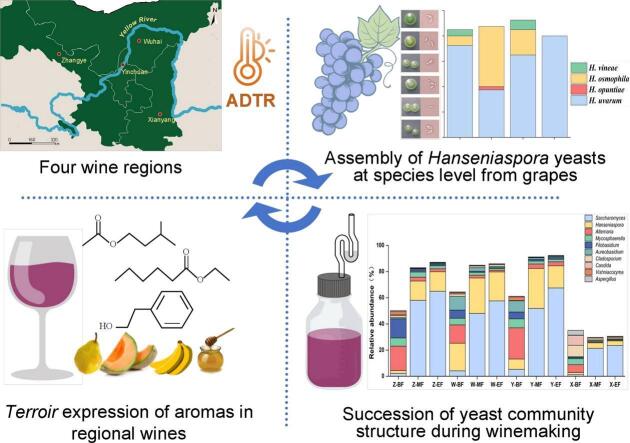

## 1 Introduction

Traditional *terroir* refers to the combination of environmental factors (e.g., climate, soil and topography) that impart unique geographical characteristics to a wine. As a modern concept expanding *terroir* in the last decade, “microbial *terroir*” focuses on the unique and geographically regionalized microbiota present in a vineyard (Gilbert et al., 2014). These microbiota mainly includes the diverse communities of fungi (especially yeast) and bacteria that are indigenous to a region, vineyard, or even grape berries, playing a crucial role in fermentation and wine development (Bokulich et al., 2016; Griggs et al., 2021). Grapes can be converted into wine through spontaneous fermentation that is naturally triggered by indigenous yeasts residing on grape berries. Non-*Saccharomyces* yeasts serve as the predominant populations initiating alcoholic fermentation; subsequently, *Saccharomyces* yeasts emerge as the dominant species to complete the fermentation process (Borren & Tian, 2020). Although spontaneous fermentation is complex and unpredictable, the resulting wines are often characterized by distinctive geographical flavors owing to region-specific microbiota in grape must (Wei et al., 2023).

Yeast communities from grapes exhibit significant variation even at small regional scales (<100 km) (Liu et al., 2021). Such variation is primarily driven by a suite of environmental factors, including regional climatic characteristics, berry sugar content, skin temperature fluctuations, and maturity levels (Gilbert et al., 2014). These factors shape community composition and structures through mechanisms of ecological filtering and niche selection (Miura et al., 2017). For instance, fungal genera such as *Hanseniaspora* and *Aureobasidium* exhibit specific spatial distribution patterns (Liu et al., 2019). The stability of these patterns across different vintages suggests that microbial distribution is not random but rather the result of long-term environmental influences. Onetto et al. (2024) further found a combined effect of vineyard precipitation gradients and temperature variations on the temporal succession of fungal communities. Previous efforts highlight the critical roles of regional moisture and temperature in niche differentiation and the stability of community assembly.

Yeasts are the key microorganisms determining the formation of wine aromas, in particular, the biosynthesis of esters that impart fruity and floral odors (Englezos et al., 2022). Variations in the abundance and distribution of yeast species during fermentation can impact aroma outcomes in resulting wines. *Hanseniaspora*/*Kloeckera* genus is the main fungal group present in grape must and spontaneous fermentation, which makes it an important model yeast species for investigating microbial heterogeneity across different regions (Liu, Zhao, et al., 2025; López-Enríquez et al., 2023). Fermentation with *H. uvarum* can significantly increase the concentrations of acetate esters and fatty acid ethyl esters in wines (Hu et al., 2018). *H. vineae* and *H. opuntiae* possess high productivity of floral and sweet odorant 2-phenylethyl acetate (Bourbon-Melo et al., 2021; Martin et al., 2016). By using *Hanseniaspora* yeasts of four species, we recently identified their species-specific effects on nutrition competition, fermentation activity, and aroma formation during mixed fermentation with *Saccharomyces* yeast (Zheng et al., 2025). Exploring the community assembly of *Hanseniaspora* species across regions may help elucidate region-driven aroma variations, and develop enological process of indigenous yeasts for enhancing wine *terroir*.

This study aimed to investigate the community assembly patterns of *Hanseniaspora* species and their contributions to aroma production in winemaking by performing spontaneous fermentation of Cabernet Sauvignon grapes from four regions in Northwest China. Volatile profiles and aroma characteristics in final wines were analyzed. Succession of yeast community structure during winemaking was determined by high-throughput ITS sequencing, followed by culture-dependent isolation and molecular identification of *Hanseniaspora* species. Furthermore, the correlation between isolated *Hanseniaspora* species, grape characteristics, environmental factors and aroma compounds was assessed to shape a potential “region-environment-microbe-aroma” interaction pathway.

## 2 Materials and methods

### 2.1 Spontaneous fermentation and sample collection

**Fig. 1:**
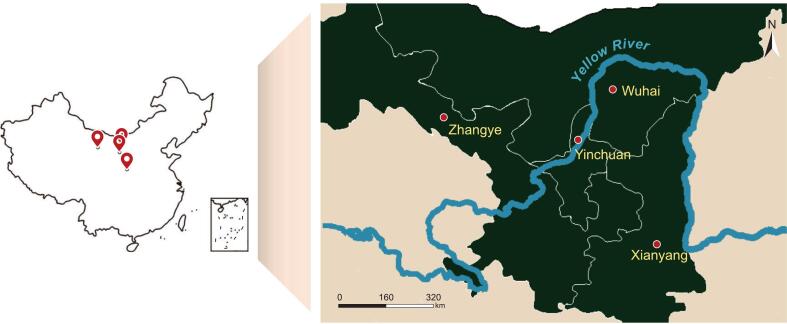
Sampling locations of Cabernet Sauvignon from four wine regions around the Yellow River in Northwest China: Zhangye (ZY), Wuhai (WH), Yinchuan (YC), and Xianyang (XY). (For interpretation of the references to color in this figure legend, the reader is referred to the web version of this article.)

Cabernet Sauvignon grapes were collected from Zhangye (ZY), Wuhai (WH), Yinchuan (YC), and Xianyang (XY), which are located in four wine-producing regions around the Yellow River Basin in Northwest China (Fig. 1). The geographical distances among these regions ranged from approximately 150 km (between YC and WH) to 900 km (between ZY and XY). Chemical properties of grape berries, as well as average diurnal temperature range (ADTR) and precipitation of four regions are shown in Table S1. Meteorological data during the month prior to harvest were obtained from the Xihe Energy Meteorological Big Data Platform (http://www.xihe-energy.com). Grapes were crushed by hand and macerated with skins at 4 °C for 18 h. To ensure homogeneous fermentation media, fermentations were performed in grape musts without skins. Grape musts were put in 500 mL glass flasks sealed with airlocks to release CO_2_, and placed in an incubator at 25 °C to initiate spontaneous fermentation. Fermentations were conducted in triplicate, without addition of sugar and sulfur dioxide. Samples were collected at three time points, i.e., before the start of fermentation (BF, initial grape must), middle stage of fermentation (MF, 50% sugar was consumed), and end point of fermentation (EF, residual sugar <2 g/L). Yeast cells in fermenting samples were obtained by centrifugation at 4 °C, 8000*g* for 5 min, frozen by liquid nitrogen, and stored at −80 °C.

### 2.2 DNA extraction and sequencing

#### 2.2.1 DNA extraction

Yeast samples were sent to Gene Denovo company (Guangzhou, China) for DNA extraction and sequencing. HiPure Soil DNA Kits (Magen, Guangzhou, China) were used to extract fungal genomic DNA according to methods documented on the kit. After extraction, NanoDrop 2000 (Thermo Scientific, USA) and Agarose Gel Electrophoresis instrument DYY-6C (Beijing Liuyi Biotechnology Co., Ltd) were utilized to examine the purity and integrity of the extract. The ITS2 region is a fungal DNA barcode with high taxonomic resolution (Bakker, 2018). Specific primers ITS3_KYO2 (GATGAAGAACGYAGYRAA) and ITS4 (TCCTCCGCTTATTGATATGC) with barcode were utilized to amplify the ITS2 region. The ITS3_KYO2 primer improves fungal specificity and reduces non-fungal amplification, making it suitable for complex environmental samples (Asemaninejad et al., 2016). PCR amplification was executed in triplicate in a total of 50 μL reaction system containing 10 μL of 5 × Q5 reaction buffer, 10 μL of 5 × Q5 high GC enhancer, 0.2 μL of Q5 high-fidelity DNA polymerase, 1.5 μL of 2.5 mM dNTPs, 1.5 μL of 10 μM each primer, 50 ng of template DNA and moderate volume of double distilled water. The target region was amplified with a procedure of 95 °C for 5 min, followed by 30 cycles at 95 °C for 1 min, 60 °C for 1 min, then 72 °C for 1 min and a final extension at 72 °C for 7 min. PCR products were first extracted from 2% agarose gels and purified using the AxyPrep DNA Gel Extraction Kit (Axygen Biosciences, Union City, CA, U.S.) according to the manufacturer's instructions, and finally quantified using ABI StepOnePlus Real-Time PCR System (Life Technologies, Foster City, USA).

#### 2.2.2 DNA sequencing

The purified DNA products were pooled in equimolar amounts and subjected to paired-end sequencing (PE250) on the Illumina platform. Raw reads were filtered using FASTP (v0.18.0) to obtain high-quality sequences. FLASH (v1.2.11) was employed to merge the reads into raw tags, and then filtered under specific criteria to yield clean tags. Subsequently, UPARSE 9.2.64 pipeline was used to cluster these clean tags into operational taxonomic units (OTUs) with similarity ≥97%. For further analysis, chimeric tags were excluded by UCHIME algorithm. And the highest-abundance tag sequence was selected as the representative sequence within each cluster. Finally, the representative sequence was classified on the basis of SILVA database 138.1 with the confidence threshold value of 0.8.

### 2.3 Isolation and identification of *Hanseniaspora* strains

*Hanseniaspora* strains were isolated from the initial grape must by culture-dependent method. Grape must samples were inoculated on a Wallerstein Laboratory (WL) nutrient agar added with 100 mg/L chloramphenicol to avoid bacteria. According to the typical colony color and morphology of *Hanseniaspora* yeasts (Martin et al., 2018), isolates characterized by both “green, smooth” colonies and lemon-shaped cells were selected and purified using WL nutrient agar. Purified isolates were subsequently grown in yeast extract peptone dextrose (YPD) medium (10 g/L yeast extract, 20 g/L peptone, 20 g/L glucose, and unadjusted pH) at 28 °C prior to DNA extraction. The genomic DNA was extracted by TIANamp Yeast DNA Kits (TIANGRN®), and sent to AuGCT DNA-SYN Biotechnology (Beijing, China) for sequencing of the 26S rDNA D1/D2 domain. Primers NL1 (5′-CATATCAATAAGCGGAGGAAAAG) and NL4 (5′-GTCCGTGTTTCAAGACGG) were selected to amplify the target DNA (Shibayama et al., 2024). Sequences were analyzed on NCBI (BLAST: Basic Local Alignment Search Tool (nih.gov).

### 2.4 Analysis of non-volatile compounds

The concentrations of glucose, fructose, ethanol, and acetic acid in the final wine samples were determined using a high-performance liquid chromatography system (HPLC, Alliance® 1260 Infinity II, Agilent Technologies, USA). Chromatographic separation was performed on an HPX-87H column (300 × 7.8 mm; Bio-Rad, USA) maintained at 60 °C. The mobile phase consisted of 5 mmol/L sulfuric acid solution at a flow rate of 0.5 mL/min, with an injection volume of 5 μL. Sugars and ethanol were detected using a refractive index detector (RID, G7162A; Agilent Technologies, USA) at 45 °C, while acetic acid was quantified using a diode array detector (DAD, G7115A; Agilent Technologies, USA) at 210 nm. Target compounds were identified by comparing their retention times with those of pure standards and quantified using calibration curves prepared from standard solutions of known concentrations.

The total titratable acidity (TA) was determined by acid-base titration using 0.05 mol/L sodium hydroxide (NaOH) as the titrant and phenolphthalein as the indicator. The pH value was measured directly using a pH meter (Ohaus, China).

### 2.5 Analysis of volatile compounds

Volatile aroma compounds in the final wine samples were determined using headspace solid-phase microextraction coupled with gas chromatography–mass spectrometry (HS-SPME-GC/MS), following the method described by Zhao et al. (2022). The GC–MS system (Shimadzu Corporation, Kyoto, Japan) equipped with a DB-WAX chromatographic column (60 m × 0.25 mm i.d., 0.25 μm film thickness, Agilent J & W, USA) was utilized. Each headspace vial containing a mixture of 2 mL of sample, 2 g of NaCl, 20 μL of 2-octanol (internal standard, 16 mg/L), and 6 mL of pure water was incubated in an autosampler (AOC-6000, Shimadzu) at 40 °C for 15 min under 600 rpm agitation, and then volatile compounds were extracted by a SARR11-DVB/CWR120/20 fiber (20 mm × 1.1 mm o.d., 120 μm phase thickness, PAL System, Switzerland) for 30 min. The completed extraction was desorbed at 230 °C in the GC infuser for 5 min. Helium was chosen as carrier gas with a flow rate of 1.5 mL/min. The temperature of the GC program was kept at 40 °C for 3 min, then increased to 160 °C at the rate of 4 °C/min, and finally, it was increased to 220 °C at the rate of 7 °C/min and held for 8 min. Temperatures of the MS ion source and connector were installed at 200 °C and 220 °C, respectively. The data was gathered and scanned at a range of 35–350 amu every 0.2 s intervals. A qualitative analysis of volatile compounds was performed by GC–MS solution according to the retention time of relevant standards and the NIST 17 library. A quantitative analysis was conducted based on standard curves established under the same analytical conditions.

### 2.6 Sensory analysis of aroma characteristics

Wine aromas were assessed by 12 panelists (6 males and 6 females), who had been trained for six months with a 54-wine aroma kit (Le Nez du Vin®, France) to improve their olfactory sensitivity. Each wine sample was placed in a black glass, and panelists rated the aroma intensity on a five-point scale (1-weak, 2-slightly weak, 3-medium, 4-slightly intense, 5-intense). The final aroma characteristics were quantified using the “modified frequency” (*MF*) method (Hu et al., 2018).

$$\mathrm{MF}\%=\sqrt{F\%I\%}$$

where *F* is the detection frequency in the percentage of the total number of panelists (“12”), and *I* is the mean intensity in the percentage of the maximum possible intensity (“5”).

Ethical statement: All sensory assessors involved in this study were affiliated with Northwest A&F University. According to institutional policy, this type of research does not require formal ethical review by the Institutional Review Board. All participants were informed that their participation was entirely voluntary and that they could withdraw at any time without any consequences. Informed consent was obtained from all participants in accordance with the 1975 Declaration of Helsinki. Furthermore, all collected data were anonymized and coded to ensure confidentiality.

### 2.7 Statistical analysis

Alpha diversity including Shannon, Chao1 and Simpson were calculated in QIIME (version 1.9.1), and principal coordinate analysis (PCoA) was performed to evaluate the distribution patterns of samples based on *β*-diversity calculated by the Bray-Curtis distance with the LabDSV R package. All the values were shown as mean ± standard deviation. SPSS 26.0 software (SPSS Inc., Chicago, IL, USA) was used for one-way ANOVA, and the Duncan test (*p* < 0.05) was used for paired comparison of the data among the same group. Origin 2021 (OriginLab Corporation., Northampton, MA, USA) was applied to draw figures. The correlation of environmental factors and *Hanseniaspora* species was performed on the Tutools platform (http://www.cloudtutu.com) based on Spearman analysis. The correlation model between volatile compounds and *Hanseniaspora* yeasts was established by partial least squares regression (PLSR) using Unscrambler 9.7 (Camo, Trondheim, Norway).

## 3 Results

### 3.1 Volatile profiles analysis

The general physicochemical parameters of the final Cabernet Sauvignon wines are presented in Table S2. All wines completed alcoholic fermentation, resulting in residual sugar content below 1 g/L. A total of 35 volatile compounds were identified in wines from the four regions, consisting of 12 higher alcohols, 3 volatile fatty acids, 5 acetate esters, 6 fatty acid ethyl esters (FAEEs), 4 other esters, 4 terpenes, and 1 norisoprenoid (Table 1). Among all identified volatiles, higher alcohols represented the most abundant chemical class, followed by esters and fatty acids. Based on odor activity values (OAV) calculations, 20 aroma compounds presented OAV > 0.1 across all four regions. Among them, isobutyl alcohol, isoamyl alcohol, 2-phenylethanol, ethyl acetate, isoamyl acetate, ethyl butyrate, ethyl hexanoate, ethyl octanoate, and *β*-damascenone showed OAV > 1, indicating their strong contributions to the sensory characteristics of the wines.

**Table 1:** Volatile compounds in spontaneously fermented wines from four wine regions (μg/L).

Compounds		ZY	WH	YC	XY	OT^a^	OAV^b^	Odor descriptor^c^
*Higher Alcohols*		366,444 ± 15,231 b	391,892 ± 33,086 b	**476,541 ± 6091 a**	342,628 ± 26,316 b			
1	Isobutyl alcohol	40,463 ± 164 d	**73,954 ± 1336 a**	65,086 ± 212 b	56,765 ± 3705 c	40,000^[1]^	1.0–1.8	fusel alcohol
2	1-Butanol	**7798 ± 1504 a**	3898 ± 278 b	1972 ± 493 b	2218 ± 516 b	150,000^[1]^	<0.1	banana, fusel
3	Isoamyl alcohol	273,560 ± 11,070 b	266,612 ± 30,583 b	**346,859 ± 3843 a**	232,988 ± 20,309 b	30,000^[1]^	8.9–12	whiskey, polish
4	1-Hexanol	410 ± 63 d	985 ± 54 b	861 ± 41 c	**2433 ± 26 a**	8000^[2]^	0.05–0.3	green, fruity
5	1-Heptanol	**35 ± 3 ab**	27 ± 0 c	**37 ± 4 a**	29 ± 0 bc	200–300^[1]^	0.1–1	lemon, citrus
6	2-Nonanol	0.36 ± 0.01 b	0.35 ± 0.00 b	**0.45 ± 0.04 a**	0.36 ± 0.01 b	70^[4]^	<0.1	fatty, green
7	1-Octanol	**1.44 ± 0.01 ab**	**2.47 ± 0.94 a**	0.31 ± 0.35 b	**2.68 ± 0.31 a**	900^[1]^	<0.1	citrus, rose
8	1-Nonanol	0.55 ± 0.03 b	**2.85 ± 0.43 a**	0.65 ± 0.00 b	0.57 ± 0.05 b	600^[1]^	<0.1	fatty, floral
9	(Z)-6-Nonen-1-ol	0.40 ± 0.03 b	**1.85 ± 0.19 a**	0.54 ± 0.01 b	0.46 ± 0.03 b	1^[5]^	0.4–1.8	honeydew, watermelon
10	1-Decanol	**0.52 ± 0.04 ab**	**0.60 ± 0.04 a**	0.38 ± 0.02 c	0.47 ± 0.05 b	400^[1]^	<0.1	fatty, fruity, rose
11	2,3-Butanediol	9269 ± 785 b	**11,291 ± 345 a**	9894 ± 635 b	3574 ± 174 c	15,000^[6]^	0.1–1	fruity, creamy, buttery
12	2-Phenylethanol	34,906 ± 1633 c	35,117 ± 470 c	**51,830 ± 859 a**	44,616 ± 1571 b	14,000^[1]^	2.5–3.7	floral, rose
*Volatile Fatty Acids*		1403 ± 102 b	651 ± 28 c	775 ± 55 c	**1669 ± 149 a**			
1	Hexanoic acid	**400 ± 19 a**	220 ± 2 b	242 ± 12 b	**372 ± 19 a**	420^[1]^	0.1–1	fatty, cheese
2	Octanoic acid	593 ± 53 b	272 ± 14 c	274 ± 7 c	**748 ± 67 a**	500^[1]^	0.5–1.5	fatty, rancid
3	Decanoic acid	410 ± 30 b	159 ± 12 d	259 ± 36 c	**549 ± 63 a**	1000^[1]^	0.1–1	fatty, rancid
*Acetate esters*		46,337 ± 699 b	**72,956 ± 930 a**	**69,142 ± 1529 a**	31,207 ± 2086 c			
1	Ethyl acetate	45,448 ± 669 c	**71,607 ± 803 a**	67,755 ± 1486 b	30,478 ± 2014 d	7500^[1]^	4.1–9.5	fruity
2	Isobutyl acetate	**103 ± 12 a**	61 ± 10 bc	65 ± 5 b	38 ± 4 c	1600^[1]^	<0.1	fruity, apple, banana
3	Isoamyl acetate	735 ± 17 b	**1131 ± 98 a**	**1199 ± 25 a**	588 ± 58 c	30^[1]^	20–40	banana
4	Hexyl acetate	25 ± 0 c	**32 ± 2 ab**	29 ± 0 bc	**36 ± 4 a**	670^[3]^	<0.1	fruity, floral
5	2-Phenylethyl acetate	26 ± 1 d	**125 ± 16 a**	94 ± 12 b	67 ± 5 c	250^[1]^	0.1–1	fruity, honey, floral
*Fatty Acid Ethyl Esters*		**1234 ± 64 a**	702 ± 42 c	796 ± 68 bc	960 ± 59 b			
1	Ethyl butyrate	**253 ± 0 a**	128 ± 4 c	167 ± 20 b	148 ± 3 bc	20^[1]^	6.4–13	strawberry, apple
2	Ethyl hexanoate	**418 ± 37 a**	184 ± 3 c	212 ± 31 c	342 ± 28 b	14^[1]^	13–30	green apple
3	Ethyl octanoate	**336 ± 17 a**	225 ± 19 c	231 ± 14 bc	268 ± 16 b	5^[1]^	45–67	fruity
4	Ethyl nonanoate	66 ± 0 b	**68 ± 0 a**	66 ± 0 b	66 ± 0 b	1300^[1]^	<0.1	soapy, cognac
5	Ethyl decanoate	**102 ± 5 a**	60 ± 8 b	73 ± 3 b	**95 ± 10 a**	200^[1]^	0.1–1	fruity
6	Ethyl laurate	**59 ± 5 a**	37 ± 8 b	47 ± 0 b	41 ± 2 b	1500^[1]^	<0.1	floral, soapy, creamy
*Other Esters*		**20,466 ± 499 a**	**17,794 ± 2116 ab**	3884 ± 553 c	16,165 ± 1285 b			
1	Isoamyl octanoate	**21 ± 0 a**	21 ± 0 b	20 ± 0 b	**22 ± 0 a**	125^[2]^	0.1–1	pineapple, sweet
2	Ethyl lactate	**19,693 ± 469 a**	**17,314 ± 2109 ab**	3647 ± 543 c	16,003 ± 1265 b	146,000^[1]^	<0.1	fruity, buttery
3	Diethyl succinate	**742 ± 30 a**	449 ± 6 b	207 ± 10 c	130 ± 18 d	1,250,000^[3]^	<0.1	cooked apple
4	Ethyl 2-phenylacetate	**10 ± 0 a**	**10 ± 0 a**	**10 ± 0 a**	**10 ± 1 a**	650^[3]^	<0.1	floral, honey
*Terpenes*		**66 ± 6 ab**	43 ± 10 b	**90 ± 17 a**	**66 ± 7 ab**			
1	*α*-Terpineol	**2.80 ± 0.20 a**	**4.00 ± 0.50 a**	**4.20 ± 1.30 a**	**3.51 ± 0.40 a**	250^[2]^	<0.1	lemon, citrus, floral
2	(*E*)-Nerolidol	**37 ± 4 ab**	17 ± 8 b	**46 ± 11 a**	23 ± 2 b	1000^[7]^	<0.1	green, floral, fruity
3	Linalool	5.12 ± 0.23 bc	4.59 ± 0.68 c	**7.41 ± 0.31 a**	5.99 ± 0.36 b	25^[2]^	0.1–1	orange, floral, terpenic
4	*β*-Citronellol	21 ± 2 b	17 ± 1 c	**32 ± 4 a**	**32 ± 3 a**	100^[3]^	0.1–1	floral, fruity, citrus
*Norisoprenoids*		1.68 ± 0.02 d	2.30 ± 0.07 c	3.66 ± 0.04 b	**5.21 ± 0.38 a**			
1	*β*-Damascone	1.68 ± 0.02 d	2.30 ± 0.07 c	3.66 ± 0.04 b	**5.21 ± 0.38 a**	0.05^[2]^	33–104	woody, herbal, floral

Data are mean values ± standard deviation. Values within the same row followed by different lowercase letters indicate a significant difference (*p* < 0.05) determined by Duncan's multiple range test. The highest concentration is presented in bold font.

Refers to ^[1]^(Hu et al., 2018), ^[2]^(Ferreira et al., 2000), ^[3]^(Wang et al., 2016), ^[4]^(Lan et al., 2022), ^[5]^(Kourkoutas et al., 2006), ^[6]^(Liu, Hao, et al., 2025) and ^[7]^(Wang et al., 2024).

a Odor threshold.

b Concentration / Odor threshold.

c Odor descriptors are obtained from The Good Scents Company (http://www.thegoodscentscompany.com).

**Fig. 2:**
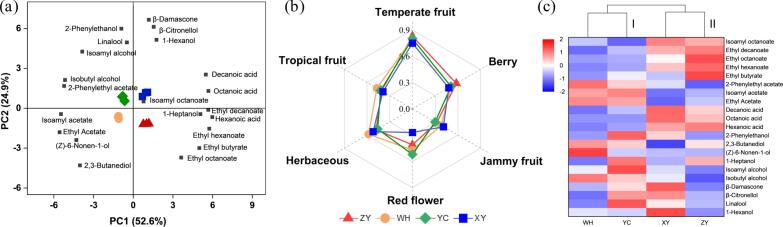
Aroma profiles of the spontaneous fermentation wine samples. (a) PCA of volatile compounds with OAV > 0.1. (b) Sensory evaluation of aroma characteristics. (c) Heat map and hierarchical clustering analysis of volatile compounds with OAV > 0.1.

Region-specific differences in volatile composition were also evident. Wines from YC region were characterized by the highest concentrations of isoamyl alcohol, 2-nonanol, 2-phenylethanol, and linalool. XY region wines exhibited the lowest levels of ethyl acetate and isoamyl acetate, whereas the total acetate ester content in WH region was 2.3-fold higher than XY region. ZY region contained the greatest abundance of fatty acid ethyl esters (FAEEs), while wines from WH displayed the opposite trend with notably lower levels. PCA further revealed clear regional differentiation in aroma profiles (Fig. 2a). Wines from different origins were positioned in distinct PCA quadrants, reflecting pronounced aromatic divergence driven by the differing volatile compositions. The aroma attribute radar plots (Fig. 2b) showed that temperate fruit was the dominant trait (*MF* > 0.6) in all the samples. Wines from YC region displayed more intense red flower and berry notes, whereas XY region wines were characterized by stronger jammy fruit and notably weaker floral expression. Wines from WH region exhibited higher herbaceous aroma. These results confirmed that Cabernet Sauvignon wines from different regions have distinct aromatic differences.

Heatmap analysis of volatile compounds with OAV > 0.1 revealed two distinct clusters (Fig. 2c). Wines from WH and YC regions clustered together due to their higher concentrations of acetate esters (ethyl acetate, isoamyl acetate, and 2-phenethyl acetate) and isobutyl alcohol. In contrast, wines from XY and ZY regions were characterized by elevated levels of fatty acid ethyl esters (ethyl decanoate, ethyl octanoate, and ethyl hexanoate), medium-chain fatty acids (hexanoic, octanoic, and decanoic acids), and increased amounts of isoamyl octanoate. Several volatile compounds displayed pronounced region-specific differences. For instance, wines from ZY, WH, YC and XY regions showed the highest concentrations of ethyl butyrate, (Z)-6-nonen-1-ol, isoamyl alcohol and 1-hexanol, respectively.

### 3.2 Fungal community dynamics during the spontaneous fermentation of wine

**Fig. 3:**
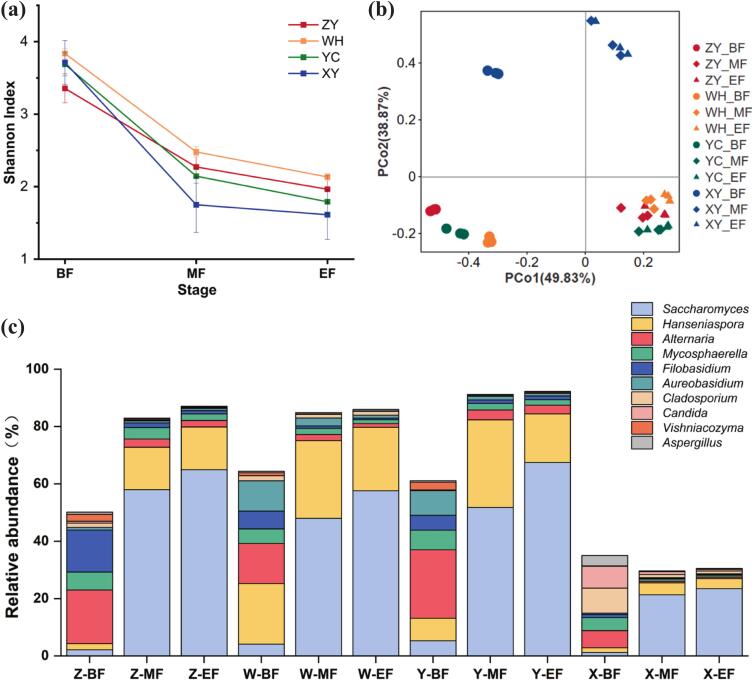
Fungal diversity and community dynamics during the spontaneous fermentation of Cabernet Sauvignon wines from four regions in Northwest China. (a) Alpha diversity measured by the Shannon index. (b) PCoA based on fungal community composition. (c) Relative abundance of the top 10 dominant fungal genera during the fermentation process.

The variations in the fungal communities during spontaneous fermentation in ZY, WH, YC, and XY are presented in this study. After quality filtering, a total of 4, 274, 138 ITS reads (with an average of 118,726) were obtained from 36 samples, accounting for 92.61% of the raw reads, respectively. The total reads clustered into fungal 896 OTUs, respectively. Table S3 provides a detailed listing of the alpha diversity estimation for all samples during the fermentation process. The fungal community diversity of four regions decreased significantly during the fermentation process (Fig. 3a). As shown in PCoA analysis, the first two principal coordinates account for 88.70% of the total variance (Fig. 3b). All the samples from BF (before the start of fermentation) stayed around the negative part of PCo1, while samples from both MF (middle stage of fermentation) and EF (end point of fermentation) were located in the positive part. Samples from ZY, WH, and YC were distributed in the negative part of PCo2, as opposed to XY samples in the positive part. Therefore, fungal communities were more similar at the middle and end stages of fermentation. Meanwhile, the fungal communities of XY region samples differed from other three regions.

The fungal OTUs of the top 10 abundance were shown in Fig. 3c, including *Saccharomyces*, *Hanseniaspora*, *Aspergillus*, *Vishniacozyma*, *Candida*, *Cladosporium*, *Aureobasidium*, *Filobasidium*, *Mycosphaerella*, and *Alternaria*. Fungal abundance differed among the four regions. Before fermentation, the genus *Alternaria* displayed high abundance in ZY and YC regions, while the genus *Hanseniaspora* dominated in WH region. Besides, the relative abundance of *Candida* was higher in XY region than other three regions grape must. However, there were some similarities in fungal diversity community succession in four regions. The abundance of *Saccharomyces* increased rapidly as fermentation proceeded, as well as *Hanseniaspora* genus. These results indicated that *Hanseniaspora* genus was the dominant non-*Saccharomyces* yeast during spontaneous fermentation in four regions.

### 3.3 Isolation and identification of *Hanseniaspora* yeasts

**Fig. 4:**
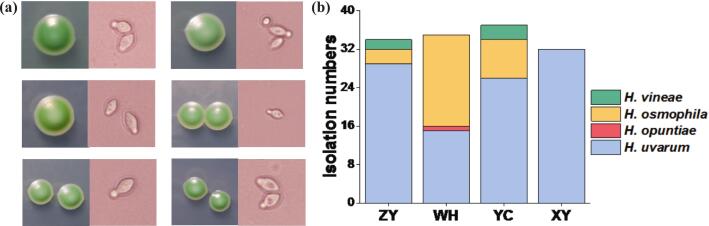
Morphological characterization and isolation numbers of *Hanseniaspora* isolates. (a) Colony morphology of *Hanseniaspora* isolates on WL nutrient agar and cell morphology. (b) Distribution and number of *Hanseniaspora* strains isolated from four regions.

*Hanseniaspora* yeast isolates exhibited “green, smooth” colonies and lemon-shaped cells (Fig. 4a). Based on DNA sequencing analysis, a total of 138 *Hanseniaspora* yeast strains were identified, including 102 strains of *H. uvarum*, 30 strains of *H. osmophila*, 5 strains of *H. vineae*, and 1 strain of *H. opuntiae*. As shown in Fig. 4b, the *Hanseniaspora* species isolated from grape juice among the four regions exhibited different proportions. In ZY and YC wine regions, strains of *Hanseniaspora* yeasts were isolated and identified mainly as *H. uvarum*, followed by *H. osmophila* and *H. vineae*. Meanwhile, in WH wine region, the most abundant *Hanseniaspora* strains were *H. osmophila* then *H. uvarum*. In the XY wine region, 32 strains of *Hanseniaspora* yeast were isolated and all identified as *H. uvarum* (Fig. 4b). These results suggested that *H. uvarum* is the most widely distributed *Hanseniaspora* species in grape juice. In addition, a higher abundance of *H. osmophila* was isolated from WH and YC wine region compared to ZY region, while *H. opuntiae* strain was isolated and identified only in WH region.

**Fig. 5:**
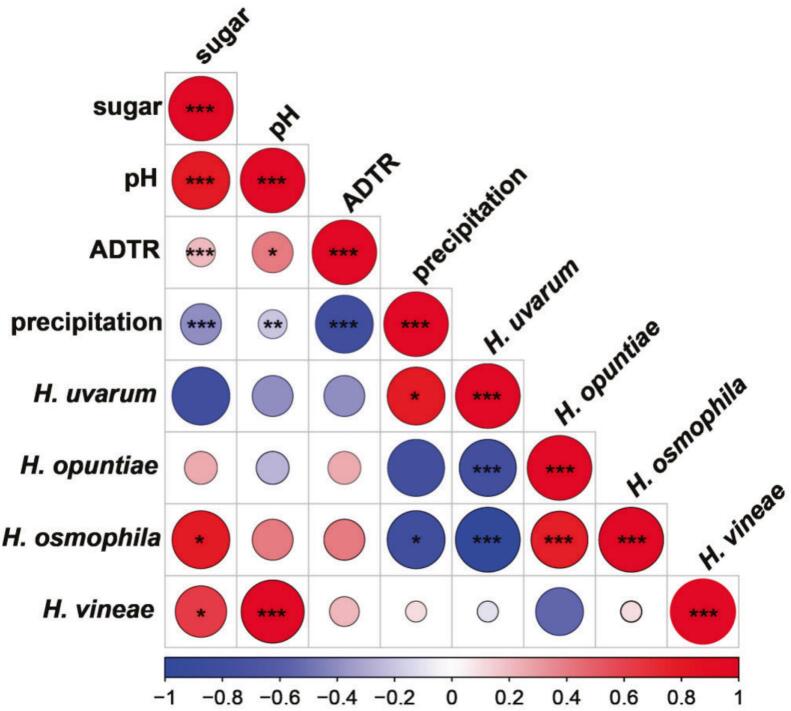
Correlation analysis between *Hanseniaspora* species, grape characteristics, and environmental factors. ADTR: average diurnal temperature range during the month prior to grape harvest. The color gradient represents the correlation coefficient, where red indicates positive correlation and blue indicates negative correlation. * *p* < 0.05, ** *p* < 0.01, and *** *p* < 0.001. (For interpretation of the references to color in this figure legend, the reader is referred to the web version of this article.)

Correlation heatmap analysis revealed that most environmental factors significantly affected the sugar content and acidity of grape berries. ADTR during the month prior to grape harvest exhibited significant positive correlation with sugar content and pH value of grape berries, while precipitation showed opposite results (Fig. 5). Furthermore, the sugar content of grape berries affected the abundance of *Hanseniaspora* species, with both *H. osmophila* and *H. vineae* showing a positive correlation with sugar levels. In addition, the abundance of *H. vineae* was positively correlated with must pH. Meanwhile, *H. opuntiae* abundance showed a significant positive correlation with *H. osmophila* but a negative correlation with *H. uvarum*. However, *H. vineae* exhibited no significant associations with the abundances of the other three *Hanseniaspora* species.

### 3.4 PLSR analysis of volatile profiles

**Fig. 6:**
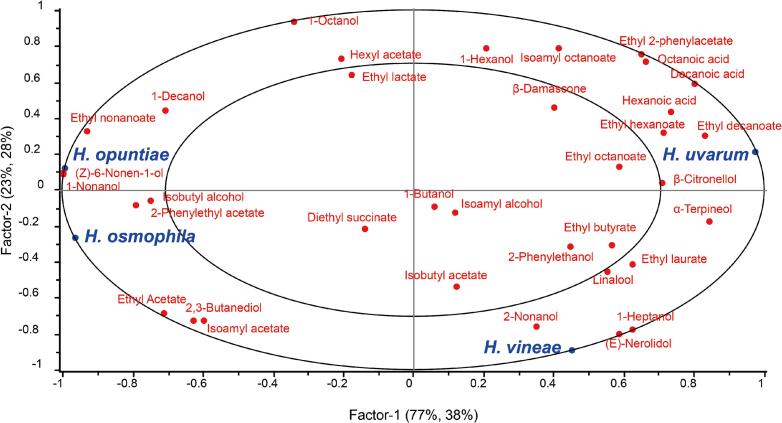
Partial least squares regression (PLSR) analysis of *Hanseniaspora* species and volatile compounds in Cabernet Sauvignon wines from four regions.

To visualize the correlation between the different *Hanseniaspora* species and volatile profiles, a PLSR analysis was performed (Fig. 6). The first two factors explained a significant portion of the variance (Factor-1: 77% X-variance, 38% Y-variance; Factor-2: 23% X-variance, 28% Y-variance), indicating a clear separation among the investigated yeast strains based on their aroma production. Notably, *H. osmophila* exhibited a distinct clustering in the third quadrant, showing a strong positive correlation with acetate esters. Specifically, this strain was closely associated with isoamyl acetate, 2-phenylethyl acetate, and ethyl acetate. *H. uvarum* was primarily characterized by medium-chain fatty acids and their corresponding ethyl esters. *H. vineae* showed a unique affinity for terpenes. Finally, *H. opuntiae* was mainly associated with higher alcohols such as 1-decanol and 1-nonanol.

## 4 Discussion

This study provides an integrated microbial and chemical perspective to explain why spontaneously fermented Cabernet Sauvignon wines from four major regions in Northwest China exhibit distinctive aroma profiles. By combining volatile profile, sensory evaluation, fungal community dynamics, and *Hanseniaspora* isolation, we disentangled how regional differences in grape chemistry, microbial ecology, and the assembly of *Hanseniaspora* species together shape wine aroma.

Spontaneously fermented Cabernet Sauvignon wines from four regions in Northwest China presented distinctive volatile profiles. Wines from ZY region were characterized by higher concentrations of fatty acid ethyl esters, while those from YC region displayed higher levels of higher alcohols. Most notably, wines from WH region were distinguished by remarkably high acetate ester content. It is worth noting that this study employed pure grape juice from cold maceration to achieve homogeneous fermentation media, and to extract varietal aroma precursors (Lukić et al., 2017). This methodological strategy ensured substrate homogeneity and minimized the influence of grape pomace on microbial distribution. Although the total number of detected compounds was limited compared to complex skin-contact fermentation, major aroma components were obtained and characterized in this study. Besides, typical volatile compounds identified in spontaneous fermentations were similar to those in Cabernet Sauvignon wines fermented with commercial yeast (Liu et al., 2016; Tang et al., 2019). This consistency underscores the stability of regional aroma profiles, which persist even under spontaneous fermentation conditions. It highlights a natural continuity between indigenous fermentation systems and commercial winemaking practices, reinforcing the concept of “microbial *terroir*” as a stable amplifier of regional distinctiveness (Chen et al., 2023).

As the fermentation progressed, *Saccharomyces* yeasts invariably dominated the final stages of fermentation, which can be attributed to their high ethanol tolerance, efficient nutrient uptake, as well as the production of antimicrobial peptides or killer toxins (Ciani & Comitini, 2015; Di Gianvito et al., 2022). However, non-*Saccharomyces* yeasts may play a crucial role by producing aroma compounds in the early stages (Wang et al., 2023). During the succession of fungal communities in spontaneous fermentation, the genus *Hanseniaspora* was identified as the dominant non-*Saccharomyces* group across all four regions (Gallo et al., 2024). Notably, by integrating high-throughput sequencing with culture-dependent method, this study further revealed species-level differentiation within the genus. Consistent with previous reports, *H. uvarum* was confirmed as the predominant non-*Saccharomyces* species in Cabernet Sauvignon musts (Padilla et al., 2016). However, the relative abundances of *H. osmophila* and *H. vineae* in ZY and YC regions were markedly elevated. These species-level differences in community assembly offer a more precise explanation for regional heterogeneity in fermentation characteristics than genus-level analyses alone. In particular, the enrichment of *H. osmophila* in WH and YC regions may be associated with its higher ethanol tolerance and adaptation to specific high-sugar environments (Valera et al., 2021).

Environmental factors influence grape quality characteristics, as well as the distribution of microorganisms on the grape skin (Yogita et al., 2021). The relatively high ADTR and low precipitation in the WH and YC regions markedly promote sugar accumulation in grape berries (van Leeuwen & Darriet, 2016). In addition, precipitation has a negative correlation with the pH value of grape, that is the grape acidity increased along with higher amount of precipitation (Costa et al., 2020). This study found that the sugar content of grape berries was significantly positively correlated with the abundances of *H. osmophila* and *H. vineae*. High-sugar conditions impose strong selective pressure on microbial communities, and these two species, owing to their enhanced osmotic tolerance and environmental adaptability, are able to outcompete other yeasts and occupy advantageous ecological niches (Lleixà et al., 2016; Valera et al., 2021). Therefore, climatic conditions may drive species turnover within the genus *Hanseniaspora* by altering the physicochemical properties of the grape substrate, thereby shaping a potential “region-environment-microbe” interaction pathway.

PLSR analysis revealed species-specific associations between *Hanseniaspora* yeasts and volatile metabolite profiles. The distinctive “red flower” and “temperate fruit” notes of wines from WH and YC regions may be attributed to *H. osmophila* and acetate esters (del Fresno et al., 2022). Previous genomic and metabolic studies have indicated that *H. osmophila* possesses highly active alcohol acetyltransferases, which efficiently catalyze the esterification of higher alcohols with acetyl-CoA (Martin et al., 2018). This may explain the significantly higher levels of acetate esters observed in high-sugar must fermentations dominated by *H. osmophila*. Excessive ethyl acetate (> 100 mg/L) can give nail polish odor in wines (Hu et al., 2018; Sumby et al., 2010). Ethyl acetate concentrations (30–72 mg/L) in the resulting wines were well below the unacceptable level, indicating that this odorant served as a contributor to fruity aromas without triggering off-flavors. *H. uvarum* is mainly associated with medium-chain fatty acids and their corresponding ethyl esters (Tristezza et al., 2016), which may contribute to the distinct sensory profiles of wines from XY and ZY regions. This species-metabolite relationship indicated that the species composition of *Hanseniaspora* is a key biological factor shaping the aroma characteristics of spontaneously fermented wines. However, the dynamics of alcoholic fermentation are fundamentally defined by the interactions between *S. cerevisiae* and non-*Saccharomyces* yeasts (Torres-Guardado et al., 2022). Although *Hanseniaspora* species are key biological factors shaping aroma characteristics, *S. cerevisiae* typically outcompetes other microbial species, eventually becoming the dominant strain (Albergaria & Arneborg, 2016). Therefore, optimizing wine quality requires a strategy where *S. cerevisiae* ensures fermentation stability as the dominant species, while *Hanseniaspora* serves as an adjunct culture to boost aromatic complexity without detrimental sensory effects.

## 5 Conclusions

This study elucidated the relationships among environmental factors, fungal communities, and aroma compounds during spontaneous fermentation of Cabernet Sauvignon across four regions in Northwest China, highlighting the crucial role of *Hanseniaspora* genus in shaping regional microbial *terroir*. Spontaneously fermented wines from the regions of ZY, WH, YC, and XY possess unique aroma profiles, which are linked to fungal diversity, in particular *Hanseniaspora* yeasts. The enrichment of *H. osmophila* in high-sugar niches and its contribution to acetate esters may provide insights into understanding aroma formation in regional wines. Future investigations are needed to develop synthetic microbial communities with these *Hanseniaspora* yeast strains for the enhancement of *terroir* expression in regional wines. Meanwhile, incorporating aroma extract dilution analysis (AEDA) and flavor dilution (FD) factor analysis will further enable the identification of key aroma-active compounds that significantly contribute to sensory profiles of wines.

## Declaration of generative AI and AI-assisted technologies in the manuscript preparation process

During the preparation of this work the authors used DeepSeek in order to assist with text refinement and to enhance the clarity of the writing. After using this tool, the authors reviewed and edited the content as needed and take full responsibility for the content of the published article.

## CRediT authorship contribution statement

**Boyuan Fan:** Writing – original draft, Methodology, Investigation, Formal analysis, Data curation. **Yuwei Chang:** Writing – original draft, Investigation. **Mengni Zheng:** Investigation, Formal analysis. **Jinding Han:** Writing – original draft, Formal analysis. **Yongsheng Tao:** Resources, Conceptualization. **Hongyu Zhao:** Writing – review & editing, Writing – original draft, Supervision, Funding acquisition, Conceptualization. **Kai Hu:** Writing – review & editing, Writing – original draft, Supervision, Project administration, Funding acquisition, Conceptualization.

## Declaration of competing interest

The authors declare that they have no known competing financial interests or personal relationships that could have appeared to influence the work reported in this paper.

## Data Availability

Data will be made available on request.

## Appendix A Supplementary data

Supplementary material The Supplementary data to this article: **Table S1.** Chemical properties of grape berries and meteorological characteristics of four regions. **Table S2.** The physicochemical parameters of final wines. **Table S3.** Alpha diversity estimation of the ITS sequencing libraries from 36 wine samples.
