# Supplementary material for: Community assembly and aroma contribution of Hanseniaspora yeasts in spontaneous fermentation of Cabernet Sauvignon grapes from four regions in Northwest China
Source: Food Chem X. 2026 Apr 23;36:103899. doi: 10.1016/j.fochx.2026.103899 (PMC13138070; doi:10.1016/j.fochx.2026.103899)
Supplement: Supplementary material — The Supplementary data to this article: Table S1. Chemical properties of grape berries and meteorological characteristics of four regions. Table S2. The physicochemical parameters of final wines. Table S3. Alpha diversity estimation of the ITS sequencing libraries from 36 wine samples. [file mmc1.docx]

The Supplementary data to this article:

**Table S1.** Chemical properties of grape berries and meteorological characteristics of four regions.

**Table S2.** The physicochemical parameters of final wines.

**Table S3.** Alpha diversity estimation of the ITS sequencing libraries from 36 wine samples.

**Appendix A. Supplementary data**

Table S1

Chemical properties of grape berries and meteorological characteristics of four regions.

| **Grape sample** | **Sugar (g/L)** | **pH** | **ADTR (°C)** | **Precipitation (mm)** |
| --- | --- | --- | --- | --- |
| ZY | 245.65 ± 3.31 b | 3.69 ± 0.02 b | 13.13 | 33.49 |
| WH | 248.68 ± 4.21 ab | 3.70 ± 0.07 b | 11.94 | 31.43 |
| YC | 255.45 ± 1.73 a | 3.86 ± 0.01 a | 11.13 | 47.78 |
| XY | 192.57 ± 3.66 c | 3.51 ± 0.02 c | 7.13 | 232.70 |

Data are mean ± standard deviation. Values displaying different letters within each column are significantly different according to the Duncan test at 95% confidence level. **ADTR:** Average diurnal temperature range during the month prior to grape harvest in 2020. **Meteorological data source:** Xihe Energy Meteorological Big Data Platform (http://www.xihe-energy.com).

Table S2

The physicochemical parameters of final wines.

| **Wine sample** | **Residual sugar (g/L)** | **pH** | **Ethanol**  **(%, v/v)** | **Acetic acid (g/L)** | **Titratable acidity (g/L)** |
| --- | --- | --- | --- | --- | --- |
| ZY | 0.94 ± 0.08 a | 3.72 ± 0.01 b | 13.34 ± 0.09 b | 0.18 ± 0.02 b | 7.65 ± 0.15 b |
| WH | 0.84 ± 0.07 a | 3.64 ± 0.02 c | 13.72 ± 0.19 a | 0.16 ± 0.01 b | 7.37 ± 0.06 b |
| YC | 0.90 ± 0.12 a | 3.79 ± 0.05 a | 13.76 ± 0.11 a | 0.26 ± 0.01 a | 7.44 ± 0.22 b |
| XY | 0.87 ± 0.10 a | 3.52 ± 0.01 d | 11.31 ± 0.08 c | 0.16 ± 0.02 b | 8.56 ± 0.43 a |

Data are mean ± standard deviation. Values displaying different letters within each column are significantly different according to the Duncan test at 95% confidence level.

Table S3

Alpha diversity estimation of the ITS sequencing libraries from 36 wine samples.

| **Region** | **Stage** | **Observed OTUs** | **Shannon** | **Chao1** | **Simpson** |
| --- | --- | --- | --- | --- | --- |
| ZY | BF | 327.33 ± 36.20 | 3.36 ± 0.20 | 395.61 ± 53.98 | 0.81 ± 0.02 |
|  | MF | 214.67 ± 37.82 | 2.27 ± 0.16 | 290.39 ± 46.18 | 0.63 ± 0.05 |
|  | EF | 211.00 ± 34.83 | 1.96 ± 0.18 | 260.08 ± 49.32 | 0.55 ± 0.05 |
| WH | BF | 356.67 ± 58.94 | 3.84 ± 0.07 | 443.78 ± 80.49 | 0.88 ± 0.00 |
|  | MF | 252.00 ± 63.10 | 2.48 ± 0.07 | 351.37 ± 92.71 | 0.69 ± 0.02 |
|  | EF | 226.33 ± 48.52 | 2.13 ± 0.05 | 303.81 ± 51.13 | 0.61 ± 0.01 |
| YC | BF | 334.67 ± 45.65 | 3.69 ± 0.16 | 379.97 ± 42.22 | 0.86 ± 0.02 |
|  | MF | 224.67 ± 46.70 | 2.15 ± 0.10 | 342.88 ± 59.49 | 0.64 ± 0.02 |
|  | EF | 231.33 ± 45.17 | 1.79 ± 0.17 | 313.29 ± 69.20 | 0.51 ± 0.05 |
| XY | BF | 317.67 ± 51.43 | 3.71 ± 0.30 | 369.91 ± 64.30 | 0.84 ± 0.02 |
|  | MF | 165.33 ± 48.44 | 1.75 ± 0.38 | 239.91 ± 88.79 | 0.52 ± 0.11 |
|  | EF | 162.33 ± 61.70 | 1.61 ± 0.34 | 222.68 ± 94.56 | 0.50 ± 0.11 |

Data are mean ± standard deviation.
